# Projectile Embolism From a Firearm Injury: A Case Report

**DOI:** 10.7759/cureus.52910

**Published:** 2024-01-25

**Authors:** Mateo Zuluaga-Gomez, Daniela Giraldo-Campillo, Daniel González-Arroyave, Robinson A Orjuela-Correa, Manuela Bedoya-Ortiz, Carlos M Ardila

**Affiliations:** 1 Emergency, Hospital San Vicente Fundación, Medellín, COL; 2 Surgery, University of Antioquia, Medellín, COL; 3 Surgery, Universidad Pontificia Bolivariana, Medellín, COL; 4 General Medicine, Universidad CES, Medellín, COL; 5 Basic Sciences, University of Antioquia, Medellín, COL

**Keywords:** emergency medical service, pulmonary circulation, cerebral venous circulation, firearms, embolism

## Abstract

Projectile embolism resulting from firearm injuries is a rare but highly lethal complication when not diagnosed early. This report presents a case of projectile embolism from a firearm injury with an unusual entry site, the cerebral venous circulation, which subsequently migrates to the pulmonary circulation with a fatal outcome. A 24-year-old male patient was admitted to a high-complexity hospital due to a gunshot wound. A plain skull computed tomography (CT) revealed a left laminar subdural hematoma and traumatic subarachnoid hemorrhage with multiple metallic fragments embedded in the skull, some penetrating the galeal sinus, with perilesional bleeding. Contrast-enhanced chest tomography showed non-thrombotic embolism of metallic fragments in the pulmonary artery for the apical segment of the left upper lobe and right intraventricular regions. Transthoracic echocardiography revealed a hyperechoic image of 3 mm in the subvalvular apparatus toward the interventricular septum. Subsequently, the patient experienced neurological deterioration with signs of cerebral edema and parieto-occipital epidural hematomas with metallic fragments and projectiles. Measures to counteract cerebral edema were initiated. Later, the patient developed mydriasis, the absence of brainstem reflexes, and experienced cardiac arrest. This report delineates a case of projectile embolism, highlighting a distinctive aspect characterized by an unusual entry point.

## Introduction

Projectile embolism from firearm injuries is a rare but highly lethal complication when not diagnosed early. While these cases are typically reported in wartime settings, the incidence of cases related to civilian social violence has increased (0.3-1.1%) [1]. It can compromise either the arterial or venous system once there is vessel disruption due to the pulsatile action of the blood vessel against the projectile. For instance, most cases are usually attributed to anterograde embolization upon entering the arterial system; however, up to 15% of cases describe a retrograde pattern upon entering the venous system [2].

Paradoxical circulation of the projectile through a right-to-left shunt within the atrial or ventricular cardiac cavities has also been described [3]. Projectile embolization typically affects the pulmonary circulation, presenting clinically as symptoms suggestive of a probable pulmonary thromboembolism, such as dyspnea, pleuritic pain, hemoptysis, or, in severe cases, hemodynamic instability. Yoon et al. reported projectile embolism localization in the pulmonary circulation in 32.2% of cases [3].

Long-term exposure to firearm projectile fragments can pose a risk for lead toxicity with various chronic manifestations, primarily affecting the central nervous system and leading to movement disorders or dementia.

This report details a case of projectile embolism from a firearm injury with an unusual entry site, the cerebral venous circulation, which subsequently migrates to the pulmonary circulation with a fatal outcome. Informed consent was obtained from the patient's relative, who consented to the publication of all images, clinical data, and other information included in the manuscript.

## Case presentation

**Figure 1:**
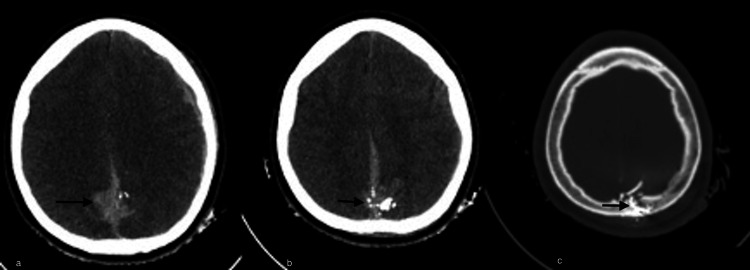
Admission plain skull computed tomography. (a) The image on the left shows bilateral parieto-occipital subarachnoid hemorrhage (arrow), and the image in the center (b) (arrow), and on the right depicts intra and extracranial projectiles (c) (arrow).

This involves a 24-year-old male patient who is admitted to a high-complexity hospital due to a gunshot wound sustained half an hour before admission. Upon admission, his blood pressure is 148/87 mmHg, mean arterial pressure is 107 mmHg, heart rate is 75 beats per minute, respiratory rate is 19 per minute, and oxygen saturation is 86%. On physical examination, dilated pupils of 4 mm are observed but are reactive to light, and limb mobility is noted, although there is paresis of the left lower limb. Due to neurological deterioration (Glasgow coma scale of 7), orotracheal intubation is performed in the emergency department. Subsequently, a plain skull computed tomography (CT) scan is conducted (Figure 1), revealing a left laminar subdural hematoma, traumatic subarachnoid hemorrhage (Figure 1a), with multiple metallic fragments embedded in the skull, and some penetrating the galeal sinus, with perilesional bleeding (Figures 1b-1c).

**Figure 2:**
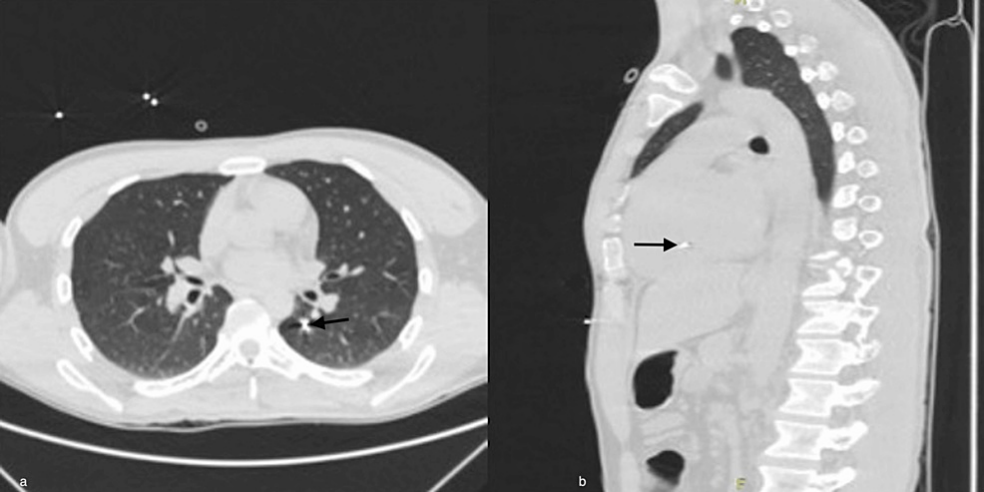
Chest computed tomography at admission. (a) The image on the left reveals a non-thrombotic embolism of metal fragments in the pulmonary artery for the apical segment of the left upper lobe and right intraventricular regions (arrow). (b) On the right, cardiac chambers of normal size are observed with metal fragments inside the right ventricular cavity (arrow).

A plain chest CT scan is also obtained (Figure 2), showing a hyperdense image suggestive of a projectile, prompting a contrasted chest CT scan, revealing a non-thrombotic embolism of metallic fragments in the pulmonary artery for the apical segment of the left upper lobe (Figure 2a) and right intraventricular region (Figure 2b).

The patient is evaluated by neurosurgery, indicating that, due to the location of deep cerebral fragments, they decide against performing fragmentectomy due to the high risk of bleeding and a fatal event. The patient is admitted to the intensive care unit (ICU) where they are kept under deep pharmacological sedation.

**Figure 3:**
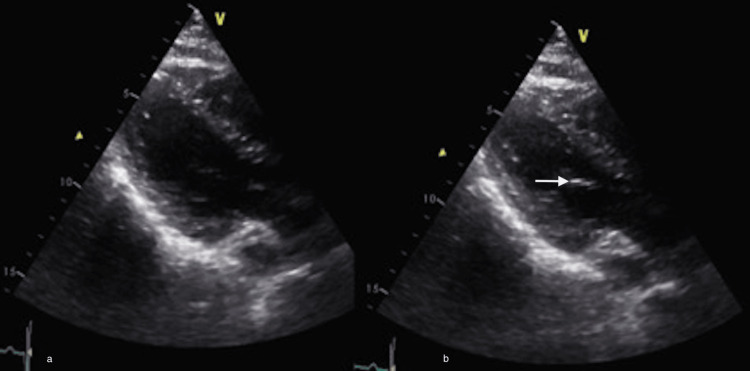
Transthoracic echocardiogram. (a) The image on the left shows the ventricle without hemodynamic alterations or changes in contractility. (b) On the right, in the subvalvular apparatus toward the interventricular septum, a hyperechoic image of 3 mm is observed (arrow).

Considering the cardiac finding, a transthoracic echocardiogram is performed (Figure 3), revealing a left ventricle with diastolic diameter within normal parameters and normal wall thickness (Figure 3a), an estimated ejection fraction by volumetric 2D and Simpson's method at 60 ± 5%. An echogenic image of 3 mm is observed in the subvalvular apparatus toward the interventricular septum (Figure 3b). The case is discussed with the cardiology service, without the benefit of surgical or endovascular interventions.

**Figure 4:**
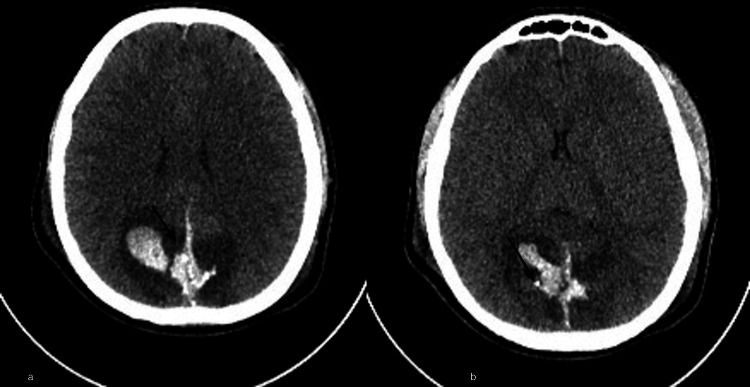
Skull tomography on the second day after admission. (a) On the left image, subarachnoid hemorrhage and bilateral parieto-occipital intraparenchymal hematomas are observed (arrow). (b) On the right, parieto-occipital epicranial hematomas with metallic shrapnel and projectiles are visible (arrow).

On the second day of ICU monitoring, the patient experiences neurological deterioration, leading to a plain skull CT scan (Figure 4), which identifies signs of cerebral edema and parieto-occipital epidural hematomas with metallic fragments and projectiles. Anti-cerebral edema measures are initiated (controlled hyperventilation and monitoring and control of intracranial pressure). On the fourth day of monitoring, the patient develops midriasis, without stem reflexes; six hours later, a cardiac arrest occurs, and resuscitation maneuvers are not performed due to the poor neurological prognosis.

## Discussion

Rich et al. presented the largest documented series of vascular trauma in the battlefield context, compiling information from the Vietnam War registry, specifically from the period between 1966 and 1971. A notable aspect of this study is the infrequent detection of projectile embolism [4]. Approximately 7,500 patients were analyzed, with only 22 of them presenting firearm projectile embolism, equivalent to 0.3% of the study. Among the types of firearm projectiles causing embolism, 19 were due to shrapnel, and three were due to the complete projectile. Arterial embolism surpassed venous embolism, with percentages of 82% and 18%, respectively. The locations where embolism was most frequently found were the cardiovascular system, followed by the pulmonary circulation. Additionally, it was relatively common in the brachial artery, popliteal artery, and carotid artery. In a lesser proportion, cases were recorded in the superficial femoral artery, internal iliac artery, hepatic artery, and radial artery [4].

In 1974, a case was reported involving a 17-year-old admitted to Cary Memorial Hospital in Caribou, Maine, due to a gunshot wound to the head - a relevant case in the medical literature concerning this location. Radiographs revealed numerous radiopaque fragments scattered throughout the right occipital-parietal area, with the main bullet shadow located near the base of the skull. After undergoing a craniotomy to extract as many fragments as possible, no attempt was made to remove the largest fragment. The patient underwent a follow-up X-ray, but the mentioned fragment was not found, prompting fluoroscopy that revealed its presence in the right ventricle. This finding proved significant in the management and monitoring of the case [5].

Cases of the same location have been reported over the years in various countries with different outcomes. One such case involved a 23-year-old patient with a gunshot wound to the posterior right shoulder, without an exit wound. Both chest X-ray and chest CT revealed the projectile in the right ventricle without affecting its function. The patient underwent conventional cardiac surgery for the removal of the projectile, resulting in a successful outcome [6].

In another report, a patient in their third decade of life with seven gunshot wounds between the chest and abdomen presented shrapnel embolization to the right atrium with a collapsed inferior vena cava [7]. The patient underwent sternotomy for the removal of the foreign body using an open technique.

Similar cases of firearm projectile embolism have been reported in Latin America. In Mexico, several cases were documented, including one with an aortocaval fistula and popliteal embolism in 1997, a patient with retrograde venovenous embolism in 1998, and two cases in Tijuana discovered during autopsy [8]. In Uruguay, a 27-year-old male patient sustained a gunshot wound to the left hemithorax at the level of the third parasternal intercostal space, without an exit wound. An intraoperative X-ray of the left lower limb confirmed the projectile lodged in the popliteal region. The popliteal artery was approached, where the impacted projectile was found, and embolectomy was performed through arteriotomy. The patient succumbed to the surgical procedure four days later [9]. In 2019, in Colombia, an 18-year-old patient with multiple gunshot wounds, including the face, neck, and right upper limb, showed a projectile in the pulmonary system on imaging. This patient had a successful outcome with a non-surgical expectant approach [10].

Projectile embolism after penetrating trauma is a rare phenomenon, occurring when a bullet enters a blood vessel, loses kinetic energy, and lodges inside it. Generally, it migrates in the direction of blood flow [8].

The entry of the projectile most commonly occurs in the venous system, and two main criteria are necessary for a projectile to become an embolus. Firstly, the projectile must have low kinetic energy to enter the vessel without traversing it. Secondly, the caliber of the projectile must be smaller than the caliber of the blood vessel, including air pellets, shotgun pellets, or multiple metal fragments, among others [11].

Multiple predisposing factors have also been described, including the force of the projectile, its caliber, and shape, the site of penetration in the vascular bed, the effect of gravity (especially in the venous system), respiratory activity, blood flow strength, the victim's position at the time of projectile entry into circulation, as well as the size and angle of blood vessels [8].

While most projectile emboli travel in the direction of blood flow, they can do so in a retrograde or even paradoxical manner due to low venous pressure [8]. Projectiles in the venous system more frequently migrate to the right ventricle than in the arterial system, and they tend to get trapped beneath the tricuspid valve or in the chordae tendineae, a mechanism found in the present case with a projectile at the ventricular level and in the pulmonary arterial system [12,13].

The clinical presentation of firearm projectile embolism varies and depends on whether the embolism is arterial or venous and its location. Arterial emboli result in symptoms in 80% of cases, with early clinical presentation due to distal ischemic complications [14].

Venous system projectile emboli are asymptomatic in 70% of cases, and symptomatic cases present manifestations mainly associated with tricuspid valve disruption, cardiac tamponade due to ventricular perforation, and pericardial effusion. Other severe manifestations such as sepsis and pulmonary embolism have been described [5,12].

The therapeutic approach to firearm projectile emboli is defined based on their location and symptoms. Surgical intervention is recommended for large intracardiac projectiles (> 5 mm), those located in the left heart chamber, involvement of hollow viscera, symptomatic patients (defined as hemodynamic instability, pain, signs of ischemia), and arterial emboli. The surgical management of venous emboli is controversial as up to 70% are asymptomatic [7,15,16].

Surgical management options include endovascular procedures or open embolus extraction. Clear indications for choosing the type of approach are not well-defined in the literature, but it is essential to recognize that minimally invasive surgery generally results in lower morbidity. Moreover, this type of intervention has been described in asymptomatic patients with compromised pulmonary circulation or surgically inaccessible locations. Therefore, a joint decision with the entire multidisciplinary team is crucial to determine the most beneficial approach for the patient [10,17,18].

Lastly, conservative management has been described for patients with emboli in the right circulation who remain asymptomatic. There is limited medical evidence regarding this type of management, and it is unclear whether periodic surveillance with imaging and the appropriate interval should be continued [17].

Other critical considerations, particularly in emergency settings, include prophylactic antibiotic coverage due to the associated risk of infection, primarily endocarditis. Anticoagulation is also recommended in pulmonary locations for a minimum duration of one year, except in cases of anemic bleeding or hemodynamically unstable patients [10,19,20].

## Conclusions

The recognition of firearm projectile embolism requires a multidisciplinary approach, where the decision for intervention should be considered based on factors such as the patient's life expectancy, hemodynamic condition, and location, among others. Currently, no guidelines are comparing surgical techniques versus interventions or advising on expectant management. Therefore, the management of these patients is determined based on clinical manifestations and findings observed in each case.
